# Comparison of censoring assumptions to reduce bias in tuberculosis treatment cohort analyses

**DOI:** 10.1371/journal.pone.0240297

**Published:** 2020-10-19

**Authors:** Meredith B. Brooks, Carole D. Mitnick, Justin Manjourides

**Affiliations:** 1 Dept. Global Health and Social Medicine, Harvard Medical School, Boston, MA, United States of America; 2 Dept. Health Sciences, Northeastern University, Boston, MA, United States of America; Texas A&M University College Station, UNITED STATES

## Abstract

**Objective:**

Observational tuberculosis cohort studies are often limited by a lack of long-term data characterizing survival beyond the initial treatment outcome. Though Cox proportional hazards models are often applied to these data, differential risk of long-term survival, dependent on the initial treatment outcome, can lead to violations of model assumptions. We evaluate the performance of two alternate censoring approaches on reducing bias in treatment effect estimates.

**Design:**

We simulate a typical multidrug-resistant tuberculosis cohort study and use Cox proportional hazards models to assess the relationship of an aggressive treatment regimen with hazard of death. We compare three assumptions regarding censored observations to determine which produces least biased treatment effect estimates: conventional non-informative censoring, an extension of short-term survival informed by literature, and incorporation of predicted long-term vital status.

**Results:**

The treatment regimen’s protective effect on death is consistently underestimated by the conventional censoring method, up to 7.6%. Models using the two alternative censoring techniques produce treatment effect estimates consistently stronger and less biased than the conventional method, underestimating the treatment effect by less than 2.4% across all scenarios.

**Conclusions:**

When model assumptions are violated, alternative censoring techniques can more accurately estimate associations between treatment and long-term survival. In multidrug-resistant tuberculosis cohort analyses, this bias reduction may yield more accurate and, larger effect estimates. This bias reduction can be achieved through use of standard statistical procedures with a simple re-coding of the censoring indicator.

## Introduction

Cox proportional hazards (PH) models are commonly used to analyze time-to-event data [1]. Although the goal of a time-to-event analysis is to follow individuals until the observation of some specified event, this is not always possible. When individuals are not observed to experience the event of interest during the study period, they are censored from the data, leaving their actual event-time unknown. A key assumption of the Cox PH model is that censor times are independent of event times conditional on the variables included in the model; referred to as non-informative censoring [2, 3]. An implication of non-informative censoring is that individuals with an unobserved event time are assumed to have the same hazard of failure as those individuals still in the cohort after that censor time. Violation of this assumption may lead to invalid treatment effect estimates.

The effects of informative censoring can be particularly prominent when Cox PH models are applied to multidrug-resistant (MDR-) tuberculosis (TB) treatment cohorts. MDR-TB is an infectious disease caused by a strain of the TB bacteria, *Mycobacterium tuberculosis* [4], that is resistant to two of the most powerful anti-TB drugs [5]. Treatment for MDR-TB is complex, including long treatment duration, numerous expensive drugs, being difficult to implement, associated with severe toxicities, and low success rates. Six mutually exclusive MDR-TB treatment outcome definitions used are based on treatment completion and bacteriologic results; they are cure, treatment completion, death, treatment default/lost to follow-up, treatment failure, and transfer out/not evaluable [6]. Cure is defined as completing treatment and having consistent culture-negative results for the final 12 months of treatment; treatment completion is defined as completing treatment but not meeting the definition of cure or treatment failure due to lack of bacteriologic test results; treatment failure is defined as two or more of five cultures recorded in the final 12 months of treatment being positive or a clinical decision to terminate treatment early due to poor response; death is defined as all-cause mortality; treatment default is defined as interruption of MDR-TB treatment for two or more consecutive months; and transfer out is defined as being transferred to another reporting facility and for whom outcomes are unknown [6]. Composite outcomes are also often used, with cure and treatment completion being classified as a “successful” outcome and the remaining four classified as “unsuccessful” outcomes. The main outcome of interest is often death.

Recommendations for conducting MDR-TB cohort analyses suggest following all MDR-TB patients in a cohort from treatment initiation until a treatment outcome definition is met, regardless of treatment duration, and performing the analyses 36 months after the last patient enrollment date in the cohort [6]. Due to the scarcity of resources in areas that experience the majority of the MDR-TB burden, and the intensity of monitoring required for TB patients during treatment, patients are rarely followed past the initial treatment outcome for longer survival times. This means that individuals enrolled earlier in the cohort who experience a non-death treatment outcome, have their observation censored from the cohort at the time of that non-death treatment outcome.

When the Cox PH model is applied, under the non-informative censoring assumption of the model, individuals with an unobserved event time, which in this case are individuals who experience one of the five non-death treatment outcomes and are not followed for longer survival, are assumed to be at equal risk of death as remaining individuals in the cohort. However, literature suggests that amongst individuals who experienced a non-death treatment outcome, the risk of death at the end of a cohort period is substantially higher for individuals experiencing unsuccessful (51.2%) versus successful non-death treatment outcomes (4.2%) [7–13]. This differential risk of death between censored observations violates the non-informative censoring assumption of the Cox PH model, potentially producing invalid effect estimates.

Although methods have been explored to adjust for informative censoring, many introduce substantial limitations in the context of MDR-TB cohort analyses. For example, inverse probability censor weighting is a method that assumes that censoring and the outcome of interest (death in this context) are independent given the set of variables included [14–17], so these individuals are weighted higher to account for the probability of censored observations remaining in the study. However, there is no way to assess this independence assumption amongst individuals who experience one of the five different non-death treatment outcomes because individuals are only followed until they meet the first outcome definition. This means that no one who experience a non-death outcome is observed to die, not because they truly do not die, but because survival time is truncated at the time they experience the non-death outcome and they are not followed longer to observe death [16–18].

Additionally, this setting can not be treated as a competing risks problem, as the five non-death outcomes do not preclude the occurrence of the primary event of interest, death due to MDR-TB [19]. Instead, these non-death outcomes are intermediary outcomes used as a proxy for longer-term outcomes because the event of interest is not yet observed due to programmatic issues leading to sub-optimal follow-up timeframes.

Without data that capture the true survival status of individuals subsequent to the initial treatment outcome, it is impossible to quantify the true magnitude and direction of the bias or to confirm that models using alternate censoring techniques produce more accurate estimates. The goal of this manuscript is to describe and evaluate a simple to implement approach to reduce the bias introduced by censoring individuals experiencing on of the five non-death outcomes. The censoring adjustments we propose can be accomplished through a simple re-coding of the cohort data and application of the standard cox proportional hazards model. Thus, we conduct a simulation study of an MDR-TB cohort to compare the performance of two alternate censoring techniques to the conventional approach to identify the method that produces the least biased treatment effect estimates.

## Materials and methods

This simulation was designed to mirror a cohort of patients who received their first treatment for MDR-TB in Lima, Peru (1999–2002) that has previously been reported [12, 13, 20–24]. No new data were collected for this analysis.

Important covariates included sex, poor nutritional status (defined as low body mass index per the Centers of Disease Control and Prevention [25] or clinical assessment of malnutrition), tachycardia, extra-pulmonary TB (EPTB), human immunodeficiency virus (HIV) coinfection, number of previous TB treatment regimens, comorbid conditions (defined as the presence of at least one of the following conditions: cardiovascular disease, diabetes mellitus, hepatitis or cirrhosis, epilepsy/seizures, renal insufficiency, psychiatric disorder, history of smoking or substance use/abuse), and number of resistant agents (resistance to the following twelve drugs or drug classes was tested: capreomycin, cycloserine, ethambutol, ethionamide, isoniazid, kanamycin or amikacin, para-aminosalicylic acid, pyrazinamide, rifampicin, streptomycin, ciprofloxacin, ofloxacin, gatifloxicin, levofloxacin, moxifloxacin [21]). Female sex, having a poor nutritional status, tachycardia, EPTB, HIV, having had two or more previous treatment regimens, and having a comorbid condition were all randomly generated from binomial distributions with probabilities equal to 0.39, 0.30, 0.30, 0.09, 0.02, 0.26, and 0.37, respectively. The number of resistant agents was generated from a normal distribution between 2 and 11, with a mean of 5.43 and a standard deviation (sd) of 1.7, and rounded to the nearest whole number. Ages, between 10 and 82, were generated from a normal distribution with a mean of 31.5 (sd: 12.0) years.

The main exposure of interest is the proportion of observed treatment time that an individual was on an aggressive treatment regimen. The observed treatment time is defined as the time from treatment initiation to the time that the initial treatment outcome was experienced. Then, the proportion of this observed time that the individual was on an aggressive regimen was calculated. An aggressive treatment regimen was defined as a regimen containing at least five likely effective drugs based on previous treatment history and baseline drug resistance pattern during the intensive phase of treatment, and at least four likely effective drugs during the continuation phase [26]. The categorization of being on an aggressive treatment regimen or not was made in retrospect after reviewing drug-susceptibility test results.This aggressive treatment regimen has previously demonstrated a reduction in mortality and recurrence of TB disease in the Peru cohort that this simulation mirrors. The proportion of time on an aggressive treatment regimen was designed to vary by age group, as observed in the Peru cohort, with a mean of 68.8% (sd: 37.8%) for adolescents (defined by the WHO as 10–19 years old [27]) and 55.0% (sd: 41.6%) for adults. To reflect this, the proportion of time on an aggressive treatment regimen was calculated in a two-step procedure. First, we produced risk scores for being on an aggressive treatment regimen as a function of the other covariates using linear regression. Next, across the estimated risk scores, the lowest 30% of adults and 19% of adolescents were assigned to have no time on an aggressive regimen, while the upper 33% of adults and 46% of adolescents were assigned to be on an aggressive regimen for their entire study time. The middle 37% of adults and 35% of adolescents had their proportion of time on an aggressive treatment regimen generated randomly with mean of 0.60 (sd: 0.18) for adults and mean of 0.65 (sd: 0.15) for adolescents.

Treatment outcomes were generated from a multinomial distribution, with probabilities of having a successful outcome (cure or treatment completion), death, treatment failure, default/transfer out (combined due to low numbers of patients who transferred out) equal to 0.66, 0.21, 0.03, and 0.10, respectively. Time to successful treatment outcomes, deaths, and treatment failure, in days, were generated from Weibull distributions to reasonably match the breakdown of event times in the Lima, Peru data using the following shape and scale parameters: 3.6 and 908, 0.9 and 419, 1.8 and 1162, respectively. Time to default and transfer out were generated from a uniform distribution between 2 and 1800 days. See S1 Appendix for R code for the data generating process.

To introduce informative censoring into the data set, as is observed in the real Peru cohort, a rejection sampling algorithm, modified from that presented by Griffin et al. [28], was used to assign outcomes and event times to covariates, conditional on age and the proportion of time on an aggressive treatment regimen. Details of the rejection sampling algorithm are as follows:

1. For each subject i, i = 1,…,N, create a vector **X**_i_ consisting of that subject’s assigned *p* covariates.
2. Define *t*_*i*_ as the event time for each individual. For *i = 1*,*…*,*N*, if *t*_*i*_ corresponds to a successful treatment outcome, *s*_*i*_ *= 1*; if it corresponds to treatment failure, *f*_*i*_ *= 1*; if it corresponds to a default/transfer out, *d*_*i*_ *= 1*. For *i = 1*,*…*,*N*, define *δ*_*i*_ as the event indicator, with *δ*_*i*_ *= 1* for death = 1, and *δ*_*i*_ *= 0* otherwise. Then, sort the *N* survival status pairs *(t*_*i*_, *δ*_*i*_*)* such that *t*_*i*_ *< t*_*i+1*_.
3. Starting from the earliest observed time, randomly assign each consecutive survival status pair *(t*_*i*_, *δ*_*i*_*)* to a covariate vector *X*_*i*_ using the following rules:
  - If *δ*_*i*_ *= 1* (i.e, *t*_*i*_ is an event [death] time), use a rejection sampler to assign the covariate vector. First, define *R*_*i*_ as the risk set for *t*_*i*_, such that *R*_*i*_ contains all individual covariate vectors that have not yet been assigned. Next, randomly select a covariate vector, **X**_j_, from *R*_*i*_ and calculate *exp(****β****’****X***_*j*_*)*/$c_{t_{i}}$ where $c_{t_{i}}$ = *max(exp(****β’X***_***i***_*))* over all covariate vectors in *R*_*i*_. Draw *U* from a uniform distribution between 0 and 1. If *U < exp(****β****’****X***_*j*_)/$c_{t_{i}}$ then assign ***X***_***i***_ to the event time, *t*_*i*,_ and the associated outcome; otherwise repeat this step.
  - If *δ*_*i*_ *= 0* and *s*_*i*_ *= 1*, use a rejection sampler to assign the covariate vector. Follow all previous steps, except if *U* ***>*** *exp(****β****’****X***_*j*_)/$c_{t_{i}}$ then assign ***X***_***i***_ to the event time, *t*_*i*_, and the associated outcome; otherwise repeat this step. If *exp(****β****’****X***_*j*_*)/$c_{t_{i}}$ = 1* then assign ***X***_***i***_ to an event time and an associated outcome by simple random sampling from *R*_*i*_ with equal probability *1/size(R*_*i*_*)*, where *size(R*_*i*_*)* is equal to the number of individuals still at risk at time t_i_.
  - If *δ*_*i*_ *= 0* and *f*_*i*_ *= 1* or *d*_*i*_ *= 1*, assign ***X***_***i***_ by simple random sampling from *R*_*i*_ with equal probability *1/size(R*_*i*_*)*.

See S2 Appendix for R code to develop the rejection sampling algorithm.

For all simulated datasets, we specified the true effects of treatment, β_1_, on time to death corresponding to a series of potential hazard ratios (HR) ranging from 0.2 to 1.4 in 0.1 increments to capture a range of likely scenarios based on published literature [20, 22]. The duration of the cohort period was defined as the maximum time from treatment start until an initial treatment outcome was observed over the entire cohort to mimic what is often used in the analysis of observational TB treatment cohorts.

Across all simulations we explored the use of three censoring techniques: equal-risk; mixed-risk; and predicted risk. The conventional, equal-risk approach assumes subjects are censored immediately after experiencing a non-death outcome, and that this censoring is non-informative. This approach assumes that all individuals experiencing a non-death treatment outcome are at equal risk of failure as those still receiving treatment. The mixed-risk censoring technique accounts for differential risk of survival among individuals who experienced one of the five non-death outcomes. Informed by the literature [7–13], we assume that individuals who experience a successful non-death outcome will be at low risk of death by the end of the study period. Thus, we assume they survive at least until the end of the study period. In contract, individuals experiencing an unsuccessful non-death outcome are assumed to be at at equal risk of dying as those still receiving treatment. The predicted-risk censoring technique incorporates a predicted end-of-study vital status for each individual as a function of their initial treatment outcome. The prediction model was developed and validated on a cohort of MDR-TB patients from Tomsk, Russia [29]. The model produced estimated survival probabilities for each individual. Then, a probability threshold was selected to maximize discriminatory properties, through use of the Youden’s index, and individuals were predicted to either remain alive at the end of the study period or to have died prior to the end of the period. Details regarding the discriminatory properties of the prediction model have been previously published [29]. Individuals predicted to survive contribute full survival time from treatment initiation until the end of the study period. Those predicted to die are censored at the time of the initial treatment outcome and assumed to be at equal risk of death as those still at-risk.

We assessed the association of treatment effect on hazard of death using Cox PH model with each of the three censoring techniques. Further, we evaluated estimates produced from each model across 13 values of the true treatment effect, β_1_. In all scenarios, model performance was evaluated based on 1000 simulated datasets, each consisting of N = 1000 subjects. See S3 Appendix for R code for model development and adjustment of the censoring assumptions.

Bias of each censoring technique, calculated as the mean difference between $\hat{\beta_{1}}$, the estimated treatment effect, and *β*_1_, the true treatment effect, across all simulations, was assessed for each model. When *β*_*1*_ *< 0* (or *log(β*_*1*_*) < 1*.*0*), a positive bias indicated underestimation of the treatment effect, while negative bias indicated overestimation. When *β*_*1*_ *≥0*, a negative bias indicated underestimation of the treatment effect, while positive bias indicated overestimation. Relative bias, $\hat{{(\beta }_{1}}-\beta_{1})/\beta_{1},$ demonstrates the relationship between the true treatment effect, *β*_*1*_, and the bias. Precision was assessed by calculating the mean of the mean squared error (MSE), $(\hat{\beta_{1}}-\beta_{1}{)}^{2}$ + variance($\hat{\beta_{1}}$), for each model. The coverage probability, defined as the percentage of 95% confidence intervals (CI) for $\hat{\beta_{1}}$ containing *β*_*1*_, and power, calculated the percentage of 95% confidence intervals for $\hat{\beta_{1}}$ containing non-null effects, were calculated for each model.

Simulations were also run on a data set with non-informative censoring. All subjects who experienced a non-death treatment outcome, *δ*_*i*_ *= 0*, were assigned a covariate vector, ***X***_***i***_, by simple random sampling from the risk set, *R*_*i*_, with equal probability *1/size(R*_*i*_*)*.

R version 3.4.1 was used for all data simulation and analyses.

### Ethics statement

Institutional Review Board approval was not requested for this study; all data were simulated and no real patient data were used.

## Results

**Fig 1:**
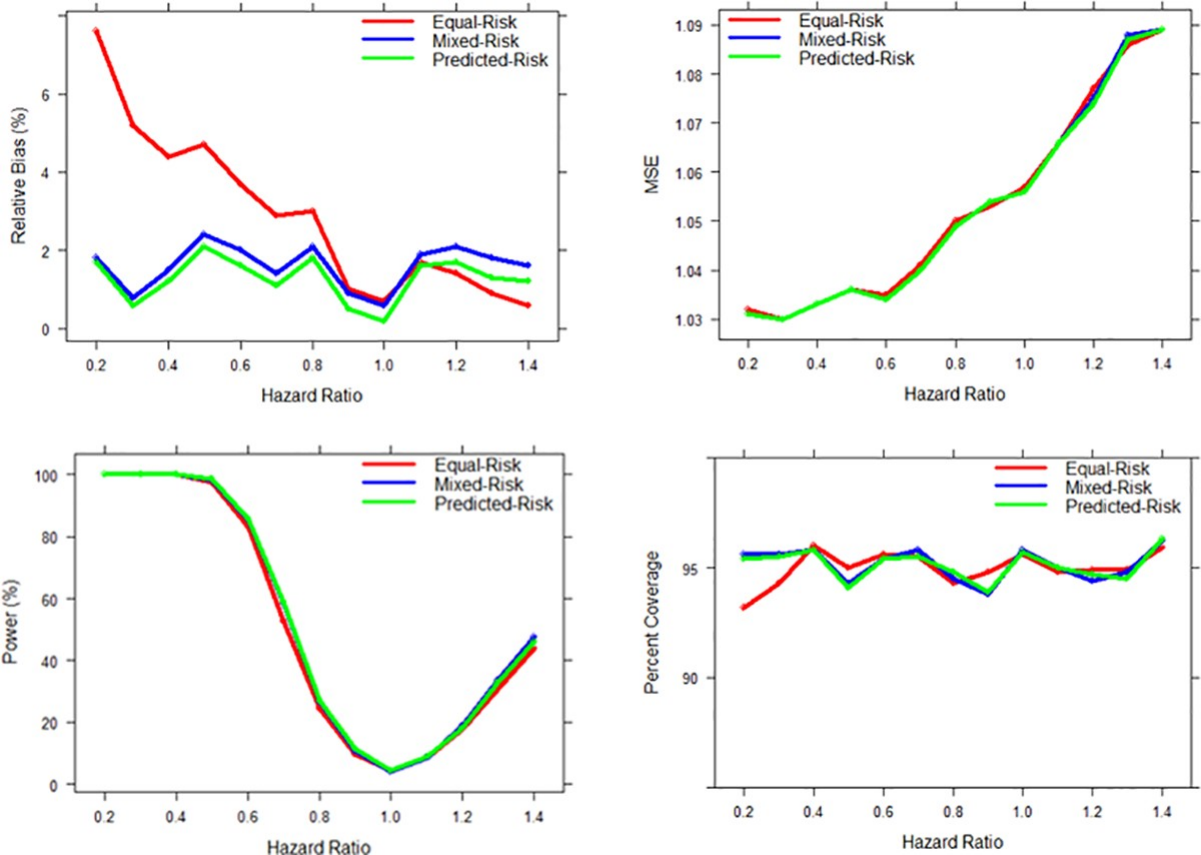
Relative bias, mean squared error, power, and 95% confidence interval coverage rates of the estimated effect of the aggressive treatment regimen by censoring technique.

For protective treatment effects (HRs less than 1.0), we observed that all bias is positive, indicating that all models underestimated the benefit of the treatment effect when informative censoring was present. Consistently, models using the predicted-risk technique were least biased, followed by the mixed-risk models, and finally the conventional equal-risk models, with relative bias increasing from 0.7 to 7.6% in parallel with stronger treatment effects. Power was consistent at the Type-1 error rate of α = 0.05 when there was no effect. When HRs are greater than 1.0, all three models overestimated the treatment effect. In these scenarios, the mixed-risk model was most biased and the equal-risk model performed better than the predicted-risk models in most scenarios. Overall, the MSEs were consistent between all censoring techniques and coverage rates hovered around 95% for all scenarios. Power increased as β_1_ moved further from the null for all models, but was slightly lower for equal-risk models in most scenarios. Table 1 shows model performance results for all β_1_ values. Fig 1 shows the model performance measures (relative bias, MSE, power, and 95% confidence interval coverage rates) for the three different censoring techniques.

**Table 1:** Model performance across treatment effect estimates.

Hazard Ratio associated with β_1_	Censoring Assumption	Relative Bias (%)	Bias	MSE	95% CI Coverage (%)	Power (%)
**0.20**	Equal-risk	7.6	0.015	1.032	93.2	100.0
	Mixed-risk	1.8	0.004	1.031	95.6	100.0
	Predicted-risk	1.7	0.003	1.031	95.4	100.0
**0.30**	Equal-risk	5.2	0.015	1.030	94.3	100.0
	Mixed-risk	0.8	0.002	1.030	95.6	100.0
	Predicted-risk	0.6	0.002	1.030	95.5	100.0
**0.40**	Equal-risk	4.3	0.017	1.033	96.0	100.0
	Mixed-risk	1.5	0.006	1.033	95.8	100.0
	Predicted-risk	1.2	0.005	1.033	95.8	100.0
**0.50**	Equal-risk	4.7	0.023	1.036	95.0	97.6
	Mixed-risk	2.4	0.012	1.036	94.3	98.3
	Predicted-risk	2.1	0.011	1.036	94.1	98.5
**0.60**	Equal-risk	3.7	0.022	1.035	95.6	83.2
	Mixed-risk	2.0	0.012	1.034	95.4	85.6
	Predicted-risk	1.6	0.010	1.034	95.4	85.9
**0.70**	Equal-risk	2.9	0.020	1.041	95.5	52.7
	Mixed-risk	1.4	0.010	1.040	95.8	58.7
	Predicted-risk	1.1	0.008	1.040	95.5	59.0
**0.80**	Equal-risk	3.0	0.024	1.050	94.3	24.2
	Mixed-risk	2.2	0.017	1.049	94.5	26.4
	Predicted-risk	1.8	0.014	1.049	94.8	26.7
**0.90**	Equal-risk	1.0	0.009	1.053	94.8	9.6
	Mixed-risk	0.9	0.008	1.054	93.8	10.7
	Predicted-risk	0.5	0.004	1.054	93.9	11.5
**1.00 (Null)**	Equal-risk	0.7	0.007	1.057	95.6	4.4
	Mixed-risk	0.6	0.006	1.056	95.4	4.6
	Predicted-risk	0.2	0.002	1.056	95.2	4.8
**1.10**	Equal-risk	1.7	0.019	1.066	94.8	8.5
	Mixed-risk	1.9	0.021	1.066	95.0	8.5
	Predicted-risk	1.6	0.017	1.066	95.0	8.7
**1.20**	Equal-risk	1.4	0.017	1.077	94.9	17.6
	Mixed-risk	2.1	0.026	1.075	94.4	18.9
	Predicted-risk	1.7	0.020	1.074	94.7	18.2
**1.30**	Equal-risk	0.9	0.011	1.086	94.9	30.4
	Mixed-risk	1.8	0.023	1.088	94.8	33.6
	Predicted-risk	1.3	0.017	1.087	94.5	32.8
**1.40**	Equal-risk	0.6	0.008	1.089	95.9	44.0
	Mixed-risk	1.6	0.022	1.089	96.2	47.4
	Predicted-risk	1.2	0.016	1.089	96.3	46.2

**MSE:** Mean squared error; **CI:** Confidence interval.

When the non-informative censoring assumption was upheld, models produced slightly biased treatment effect estimates, with no discernable pattern regarding a best performing model or a direction of bias. Table 2 shows model performance results for all β_1_ values when the non-informative censoring assumption is upheld.

**Table 2:** Model performance across treatment effect estimates with non-informative censoring assumption upheld.

Hazard Ratio associated with β_1_	Censoring Assumption	Relative Bias (%)	Bias	MSE	95% CI Coverage (%)	Power (%)
**0.20**	Equal risk	0.0	0.000	0.066	94.9	100.0
	Mixed risk	1.0	0.005	0.066	95.0	100.0
	Predicted risk	1.0	0.005	0.066	95.1	100.0
**0.30**	Equal risk	-1.3	-0.004	0.058	95.5	100.0
	Mixed risk	-0.3	-0.001	0.058	95.4	100.0
	Predicted risk	-0.3	-0.001	0.058	95.1	100.0
**0.40**	Equal risk	-1.0	-0.004	0.055	94.3	99.8
	Mixed risk	-0.3	-0.001	0.055	94.4	99.8
	Predicted risk	-0.3	-0.001	0.055	94.5	99.8
**0.50**	Equal risk	-1.8	-0.009	0.054	96.1	99.3
	Mixed risk	-1.4	-0.007	0.054	96.1	99.2
	Predicted risk	-1.4	-0.007	0.054	96.1	99.2
**0.60**	Equal risk	0.7	0.004	0.056	95.5	88.1
	Mixed risk	0.8	0.005	0.056	95.4	88.0
	Predicted risk	0.8	0.005	0.056	95.5	87.9
**0.70**	Equal risk	-0.1	-0.001	0.056	94.1	59.4
	Mixed risk	0.0	-0.000	0.055	94.1	59.6
	Predicted risk	0.0	-0.000	0.055	94.2	60.1
**0.80**	Equal risk	0.9	0.007	0.052	95.3	26.1
	Mixed risk	1.0	0.008	0.052	95.5	26.4
	Predicted risk	1.0	0.008	0.052	95.3	26.5
**0.90**	Equal risk	0.2	0.002	0.055	94.3	11.7
	Mixed risk	0.3	0.003	0.055	94.6	11.7
	Predicted risk	0.3	0.003	0.055	94.5	11.7
**1.00 (Null)**	Equal risk	-0.2	-0.002	0.055	95.3	4.7
	Mixed risk	-0.2	-0.002	0.055	94.9	5.1
	Predicted risk	-0.2	-0.002	0.055	94.9	5.1
**1.10**	Equal risk	-0.7	-0.008	0.055	93.3	9.9
	Mixed risk	-0.6	-0.007	0.056	93.5	10.4
	Predicted risk	-0.6	-0.007	0.056	93.6	10.2
**1.20**	Equal risk	0.2	0.002	0.055	96.8	19.0
	Mixed risk	0.2	0.002	0.055	96.4	18.1
	Predicted risk	0.2	0.002	0.055	96.2	18.8
**1.30**	Equal risk	-0.3	-0.004	0.053	96.2	34.5
	Mixed risk	-0.4	-0.005	0.053	96.1	34.1
	Predicted risk	-0.4	-0.005	0.053	96.0	33.5
**1.40**	Equal risk	-0.2	-0.003	0.054	94.7	52.8
	Mixed risk	-0.3	-0.004	0.053	94.9	52.4
	Predicted risk	-0.3	-0.004	0.053	95.0	52.2

**MSE**: Mean squared error; **CI**: Confidence interval.

## Discussion

Comparing the performance of Cox PH models across three different censoring techniques on simulated data sets, we find that the conventional censoring method results in biased treatment effect estimates in the presence of informative censoring. The conventional method was used in numerous analyses of the effects of the aggressive treatment regimen, meaning its benefits were potentially underestimated in previous analyses. We propose two alternative censoring techniques for application to MDR-TB cohorts that account for the differential risk of death among censored observations. While more advanced methods have been developed in other content areas, these methods are not typically applied to MDR-TB treatment cohorts, leaving these cohorts particularly susceptible to bias. These alternate techniques provide a relatively crude and direct method to adjust for the presence of informative censoring in similar MDR-TB treatment cohorts to more accurately account for the differential risk of death after the initial treatment outcome. This simulation study demonstrate that the mixed- and predicted-risk techniques can produce more accurate treatment effect estimates than the conventional model across most scenarios, with the predicted-risk model outperforming the other two.

In scenarios in which the treatment is protective against death, use of the conventional method consistently underestimates the true treatment effect in this cohort. This underestimation occurs because the conventional equal-risk assumption does not reflect the true survival experience of patients after the initial treatment outcome definition is met. Literature suggests that most individuals who experience a successful non-death initial treatment outcome will remain alive at study end [7–13], whereas the conventional method assumes that they are at equal risk of death as remaining at-risk individuals in the cohort. Thus, we observe that application of models utilizing the two alternative censoring techniques, each differentiating between risk of death for successful versus unsuccessful non-death treatment outcomes, consistently reduces bias and produces more accurate treatment effect estimates.

In scenarios in which the treatment is adversely associated with death, use of the mixed- and predicted-risk censoring techniques no longer consistently produce the least biased estimates. The extension of survival time for individuals experiencing a successful non-death treatment outcome in both the mixed- and predicted-risk techniques inherently imparts a more protective effect, with healthier individuals remaining in the study longer. If there is, in fact, a true adverse effect of the treatment regimen, these models may not produce accurate effect estimates due to the underlying assumption of increased survival and less event occurrences. In these instances, the conventional model may be more appropriate to use, or another alternative model that makes different assumptions about the longer survival of censored observations.

When the non-informative censoring assumption was upheld, no variation in the censoring assumption performed better than the others, indicating that the censor times truly were independent of the event times [2, 3]. In these scenarios, use of the conventional model seems appropriate.

This simulation study is limited by the parameters used to develop characteristics, treatment outcomes, and survival times. However, these parameters were based on a real MDR-TB cohort in which the conventional model was applied to evaluate the effect of the aggressive treatment regimen on death. In a real MDR-TB cohort, it is impossible to know the true treatment effect, resulting in an inability to assess bias. Mirroring this data and declaring true treatment effects in simulation allows for produced effect estimates to be compared to the established true effects to better understand the magnitude and direction of the bias that may have resulted in the real cohort. Although newer treatment outcome definitions have since been put in practice, we used the standardized treatment outcome definitions at the time of the initial reporting of the real cohort’s outcomes from which our simulation mirrored [6].

Results of this simulation may not be generalizable to cohorts with different distributions of patient characteristics, proportions of successful treatment outcomes or deaths, or survival times. However, it provides a framework in which to think about biased effect estimates and to demonstrate how the assumptions we make about the survival of MDR-TB patients after the initial treatment outcome, coupled with the type of analytic method being applied, can impact the results and the subsequent interpretation of results. This can be especially important for TB researchers to understand, as often times these observational cohort study analyses are what influence treatment guidelines and any bias, in either direction, may change the interpretation of results. Additionally, there are inherent issues with presenting a single hazard ratio as the measure of effect due to the assumption that this hazard ratio would remain constant overtime. In reality, it is more likely that the hazard ratio varies over specific periods of time. However, the presentation of the hazard ratio was purposefully chosen due to it being a common measure standardly reported as an effect estimate in MDR-TB treatment cohort studies and because, for the purpose of a simulation study, if provides a single estimate to compare across multiple scenarios [30].

We recognize that, while both alternative censoring techniques are found to be more accurate than the conventional method, they are also making assumptions that may not be valid across different populations, and are also more extreme assumptions than many state-of-the-art approach for handling censoring would introduce. However, due to an inability to check the assumptions of these other methods because survival time is truncated at the time of the non-death treatment outcomes, precluding the observation of death amongst the censored group, we feel they are justified. Produced estimates, however, should be interpreted with great caution and should merely serve as a benchmark for the magnitude and direction of potential bias that can be introduced when assumptions are made about survival status past the initial treatment outcome. Despite the predicted-risk models performing so well, it is based on a predictive model validated in a cohort of MDR-TB patients from Tomsk, Russia [29] that also assessed the effect of the aggressive treatment regimen, but has not been validated on the Lima cohort due to the lack of long-term survival data available.

Despite the limitations, the need to check for the presence of informative censoring remains; this study highlights the potential bias that violating this assumption can introduce and proposes simple alternative censoring techniques that can reduce this bias or, at very least, provide insight into the potential magnitude and direction of bias that is introduced.

## Conclusions

Those planning to analyze MDR-TB cohort data using Cox PH models should be aware of the potential of violating the non-informative censoring assumption and subsequent bias of treatment effect estimates due to the lack of longer-term survival status available. Adjusting the censoring technique used in the models can reduce bias and estimate treatment effects more accurately or provide evidence of bias where otherwise unclear. While the most accurate treatment effect estimates would be obtained through appropriate monitoring and follow-up of TB patients, this is often not possible in low-resource, high-TB burden settings. When survival data past the initial treatment outcome is not available, the two proposed alternative censoring approaches are simple to implement, yet crude adjustments, at the analysis stage through recoding of survival times. These may serve as useful tools to researchers who aim to use programmatic data on TB patients to gain insight into treatment effectiveness. They can provide less biased effect estimates, or a range of potential estimates, in lieu of one potentially biased estimate that may lead to incorrect interpretation of results.

## Supporting information

S1 Appendix RCode for data generation. (TEX)

S2 Appendix RCode for rejection sampling algorithm. (TEX)

S3 Appendix RCode for models and censoring. (TEX)
